# The role of melatonin as an antioxidant in the follicle

**DOI:** 10.1186/1757-2215-5-5

**Published:** 2012-01-26

**Authors:** Hiroshi Tamura, Akihisa Takasaki, Toshiaki Taketani, Manabu Tanabe, Fumie Kizuka, Lifa Lee, Isao Tamura, Ryo Maekawa, Hiromi Aasada, Yoshiaki Yamagata, Norihiro Sugino

**Affiliations:** 1 Department of Obstetrics and Gynecology, Yamaguchi University Graduate School of Medicine, Minamikogushi 1-1-1, Ube, 755-8505 Japan; 2 Department of Obstetrics and Gynecology, Saiseikai Shimonoseki General Hospital, Yasuokacho 8-5-1, Shimonoseki, 759-6603 Japan

**Keywords:** melatonin, oocyte, ovulation, reactive oxygen species, antioxidant

## Abstract

Melatonin (N-acetyl-5-methoxytryptamine) is secreted during the dark hours at night by pineal gland, and it regulates a variety of important central and peripheral actions related to circadian rhythms and reproduction. It has been believed that melatonin regulates ovarian function by the regulation of gonadotropin release in the hypothalamus-pituitary gland axis via its specific receptors. In addition to the receptor mediated action, the discovery of melatonin as a direct free radical scavenger has greatly broadened the understanding of melatonin's mechanisms which benefit reproductive physiology. Higher concentrations of melatonin have been found in human preovulatory follicular fluid compared to serum, and there is growing evidence of the direct effects of melatonin on ovarian function especially oocyte maturation and embryo development. Many scientists have focused on the direct role of melatonin on oocyte maturation and embryo development as an anti-oxidant to reduce oxidative stress induced by reactive oxygen species, which are produced during ovulation process. The beneficial effects of melatonin administration on oocyte maturation and embryo development have been confirmed by in vitro and in vivo experiments in animals. This review also discusses the first application of melatonin to the clinical treatment of infertile women and confirms that melatonin administration reduces intrafollicular oxidative damage and increase fertilization rates. This review summarizes our recent works and new findings related to the reported beneficial effects of melatonin on reproductive physiology in its role as a reducer of oxidative stress, especially on oocyte maturation and embryo development.

## Intoduction

Reactive oxygen species (ROS) are formed continuously in cells as a consequence of both oxidative biochemical reactions and external factors. While physiological levels of oxygen are necessary for cells to live, ROS such as superoxide radical (O2-), hydroxyl radical (·OH), and hydrogen peroxide (H_2_O_2_) are generated from oxygen. ROS can regulate cell function by controlling the production and activation of substances that have biological activities and by activating key downstream cell-signaling pathways [1-3]. However, surplus generation of ROS interact with lipid, protein and nucleic acid, resulting in a loss of membrane integrity, structural or functional changes in proteins, and damage in nucleic acids. Therefore, an increase in the production of ROS have detrimental effects on cell function and contributes significantly to several diseases, including those that may compromise reproduction and fertility [4].

ROS are produced within the follicle, especially during the ovulatory process. ROS play a physiological role in the process of ovulation, e.g. follicle rupture. However, an excessive amount of ROS cause oxidative stress and may damage oocyte and granulosa cells. Accumulating data have shown that ROS accelarate oocyte aging and deteriorate oocyte quality [5,6]. On the other hand, antioxidant defense systems, such as superoxide dismutase (SOD) or glutathione (GSH), are present in follicles. The balance between ROS and antioxidants within the follicle seems to be critical to the function of oocyte and granulosa cells.

Melatonin, a hormone mainly synthesized in the pineal gland, has multiple effects on a number of different physiological processes. Melatonin plays a key role in a variety of important physiological functions, including circadian rhythms [7], reproductive [8], neuroendocrine [9], cardiovascular [10], neuroimmunological [11], oncostatic actions [12]. We already reported that melatonin plays a role in lipid metabolism [13], pregnancy and parturition time [14-16], and corpus luteum (CL) function [17]. Some effects of melatonin are mediated through specific membrane receptors, but many of them seem to rely on its potential as a direct free radical scavenger, a process that requires no receptor. A growing number of studies have demonstrated that melatonin is a powerful direct scavenger of free radicals. In contrast to the majority of other known radical scavengers, melatonin is multifunctional and a universal antioxidant. The high lipophilicity and hydrophilicity of melatonin permits its rapid transfer into other organs and fluids, and melatonin can easily pass through cell membranes. Interestingly, high levels of melatonin have been found in human follicular fluid [18,19]. We already reported that human preovulatory follicular fluids contain higher concentrations of melatonin than of plasma and the melatonin concentrations in follicular fluids increased depending on follicular growth [20]. Although the physiological roles of melatonin in follicular fluid have not been understood, it is possible that melatonin is the most effective antioxidant in the follicle. The purpose of this current review is to summarize recent developments in the field of melatonin research, with a focus on how melatonin directly protects oocyte from oxidative stress within the ovarian follicle.

### Ovulation and reactive oxygen species (ROS)

ROS is locally produced during follicular rupture and may be involved in the ovulation process. Luteinizing hormone (LH) surge induces a dissolution of the basement membrane between the granulosa and theca interna layers and an expansion of the theca capillaries into the avascular granulosa cell layer to form a dense network of capillaries. Macrophages and neutrophiles are well-documented to reside in follicles; it is also well-documented that they are taken into the follicles [21,22]. Tremendous amounts of free radicals are produced within the follicle not only by macrophages and neutrophiles but also by the endothelial cells of the capillaries. Locally produced ROS seems to have an essential role on follicle rupture, and ROS also have an important role as second messengers modulating the expression of genes that govern physiological processes of oocyte maturation [23,24]. However, excess ROS can also be responsible for oxidative stress; they can damage molecules and structures of oocyte and granulosa cells within the follicle. ROS must be continuously deactivated to keep only the small amount necessary to maintain normal cell function. Follicular components, cumulus cells and the follicular fluid, may protect the oocytes from the damaging effects of ROS [25-27]. It is well recognized that endogenous antioxidant enzymes and non-enzymatic antioxidants are present in the follicles and are working to combat or reduce ROS. Failure or deficiency of these oocyte defenses could result in accumulation of ROS and the development of oxidative stress with resultant oocyte damage [28]. Additionally, ROS may be overproduced in response to several conditions, such as infections, inflammation, chemotherapy, radiation, and superovulation as an infertility therapy.

**Figure 1:**
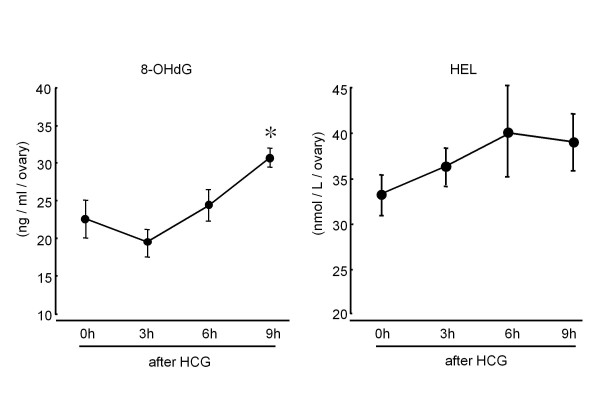
**Changes in intrafollicular concentrations of 8-hydroxy-2'-deoxyguanosine (8-OHdG) and hexanoyl-lysine adduct (HEL) during ovulation process**. Immature (3 wks) rats received a subcutaneous injection of 20 units of pregnant mare serum gonadotropin (PMSG) to stimulate the multiple follicles. 48 hrs after PMSG injection, human chorionic gonadotropin (HCG) injection was performed to induce ovulation. The ovaries were quickly removed 0, 3, 6 and 9 hr after HCG injection and were transferred to the 500 μl of 0.01 M PBS buffer. Follicular fluids were collected by puncturing ovarian follicles with a 26-gauge needle under dissecting microscope and centrifuged at 300 g for 10 min, and the supernatants were collected and stored at -80C until assay. 8-OHdG and HEL concentrations were measured using ELISA kit. Data are shown as the mean ± SEM for 4 rats. *: p < 0.05 versus 0 h, 3 h.

To assess whether the oxidative stress would develop within the follicle during the ovulation process, intrafollicular concentrations of oxidative stress markers were measured in pregnant mare serum gonadotropin (PMSG) treated immature rats (Figure 1). Intrafollicular concentration of 8-hydroxy-2'-deoxyguanosine (8-OHdG), as a DNA-related oxidative stress marker, was increased after human chorionic gonadotropin (hCG) injection. The 8-OHdG concentration was significantly increased at 9 hrs after hCG injection, just before ovulation. Intrafollicular concentration of hexanoyl-lysine adduct (HEL, a biomarker of lipid peroxidation) was also increased after hCG injection. Taking the results into consideration, ROS is physiologically produced within the follicles after LH surge, and the surplus ROS which could not be scavenged by antioxidants is responsible for oxidative stress during ovulation process. Oxidative stress may be a cause of poor oocyte quality. ROS such as superoxide radical (O2-), hydroxyl radical (·OH), hydrogen peroxide (H_2_O_2_) are known to be detrimental to the oocyte. They cause deterioration of cell membrane lipids, destroy DNA and induce two-cell block, apoptosis, and inhibition of fertilization in mouse and hamster [27,29,30]. Chao et al. [31] investigated oocyte competence after ovarian hyperstimulation in mice. Ovarian mitochondrial DNA mutation, an increase in mtDNA large scale deletions, and oxidative damage, an increase in 8-OHdG, were seen after repeated ovarian stimulation by exogenous gonadotrophin. Also, higher levels of the oxidant H_2_O_2 _have been reported in fragmented human embryos compared with non-fragmented embryos and unfertilized oocytes [6]. These results suggested that excessive oxidative stress may be a cause for poor oocyte quality.

It is well documented that antioxidant enzymes, such as (SOD), glutathione peroxidase (GPx) and catalase, and non-enzymatic antioxidants, such as vitamin E, vitamin C, glutathione, uric acid and albumin, are present in the follicles [4,5,32]. Reduced antioxidant enzyme levels, such as GPx, are reported in the follicular fluids of women with unexplained infertility [33]. Another report demonstrated that a higher level of SOD activity in follicular fluid efficiently reduced DNA damage caused by oxidative stress in porcine oocytes and cumulus cells, resulting in successful fertilization and development to the blastocyst stage after in vitro insemination; however, these abilities were interrupted by the SOD inhibitor [26]. When mice were given antioxidant supplements (vitamins C and E), an increased number of normal MII oocytes and decreased percentage of apoptotic oocytes were observed in comparison with the control group [34]. The balance between ROS and antioxidants within the follicle seems to be critical for oocytes.

### Melatonin as a free radical scavenger

Although melatonin exerts effects through its receptors, melatonin also can act as a powerful direct free radical scavenger. In 1993, melatonin was discovered to function as a direct free radical scavenger when it was shown to detoxify the highly reactive hydroxyl radical (·OH) [35,36]. Since then, many reports have confirmed the ability of melatonin to reduce oxidative stress [37,38]. In these investigations, melatonin was found to scavenge both oxygen- and nitrogen-based reactants [39,40] in several subcellular organelles [41]. Melatonin works in a variety of ways to reduce the levels of oxidative stress. It has been shown that melatonin has the capability of quenching reactive oxygen as well as reactive nitrogen species including superoxide radical (O2-), hydroxyl radical (·OH), singlet oxygen (^1^O2), hydrogen peroxide (H_2_O_2_), hypochlorous acid (HOCl), nitric oxide (NO·) and the peroxynitrite anion (ONOO-) [41-44]. Three key players are involved in ROS damage to cells: hydrogen peroxide (H_2_O_2_), superoxide radical (O2-), and hydroxyl radical (·OH). H_2_O_2 _and superoxide radicals (O2-) are thought to create less damage than hydroxyl radical (·OH), however, in the presence of free iron, specifically ferrous iron, H_2_O_2 _is converted to hydroxyl radical (Fenton reaction). Hydroxyl radical (·OH) is the most potent free radicals and is known to produce damage to all biological membranes and DNA. Melatonin can easily pass through cell membranes because of its properties of lipophilicity and hydrophilicity, and it has been demonstrated that a high levels of melatonin exist not only in cytoplasm but also beside the nucleus. The antioxidant properties of melatonin as a cell protector have been extensively studied and a previous report demonstrated that the melatonin's ability to detoxify the hydroxyl radical (·OH) was higher than well-known scavengers including vitamin C and vitamin E [37].

Not only is melatonin itself a direct free radical scavenger, but also metabolites that are formed during these interactions, i.e., cyclic 3-hydroxmelatonin, N1-acetyl-N2-formyl-5-methoxykynuramine (AFMK) and N1-acetyl-5-methoxykynuramine (AMK), are likewise excellent scavengers of reactive species [40,41,45-47]. In addition, melatonin has a high capability to detoxify ROS and suppresses the oxidative effect indirectly by enhancing the production of endogenous antioxidants. Melatonin has been stimulates activities and mRNA levels of antioxidative enzymes including (SOD), (GPx), and catalase [48,49]. Thereby, these multiple actions of melatonin protect cells from ROS-mediated lipid peroxidation, protein destruction and nuclear DNA damage [50-54].

### Melatonin and reproduction

The roles of melatonin in reproduction are focused on its direct actions in the ovary. Melatonin can pass through all cell membranes and enter all tissues because of its lipophilic property, however, it specifically concentrates in the ovary when injected systemically [55]. High levels of melatonin are found in human preovulatory follicular fluid at concentrations which are higher than serum levels [18,19]. We previously demonstrated that melatonin concentrations in the ovary showed a similar phasic variation with high levels during mid-dark and low levels during mid-light, just as in the pineal gland and serum of hamsters [56]. These concentrations were highest in proestrus, when the ovary has preovulatory follicles, during the estrus cycle. We also found the concentrations of preovulatory follicular fluid (> 18 mm) in in-vitro fertilization and embryo transfer (IVF-ET) patients are significantly higher than in small (10-12 mm) and middle follicles (15-16 mm) [20]. These results demonstrated that melatonin levels in ovarian follicles increase depending on follicular growth. We presume that the majority of melatonin found in follicular fluid enters the follicle from blood because no clear mRNA expression of NAT (the rate-limiting enzyme of melatonin) could be found in the granulosa cells of rats and humans (unpublished data), and administration of melatonin dose dependently increased melatonin concentrations in the follicle in human (data not shown). Increased melatonin in follicular fluid seems to have an important role in ovulation.

### Melatonin, oocyte quality, and embryo development

High quality oocytes produce well-developed embryos. After fertilization, ooplasm becomes the embryo cytoplasm, but the spermatozoon's participation in this process is minimal. It has been thought that the first steps of embryogenesis are controlled exclusively by maternal information present in the oocyte. For this reason, the quality of oocytes is a key factor in determining the quality of the early steps of embryo development. Oocyte maturation begins with the resumption of meiosis, and oocytes are arrested at prophase of the first meiotic division. Only fully grown oocytes can resume meiosis in response to LH surge. Oocytes pass through the first meiotic division and then become arrested at metaphase of the second meiotic division until fertilization. During this long period of meiotic maturation, oocyte accumulate molecules of mRNA, proteins, lipid and sugars as well as oxidative stress.

**Figure 2:**
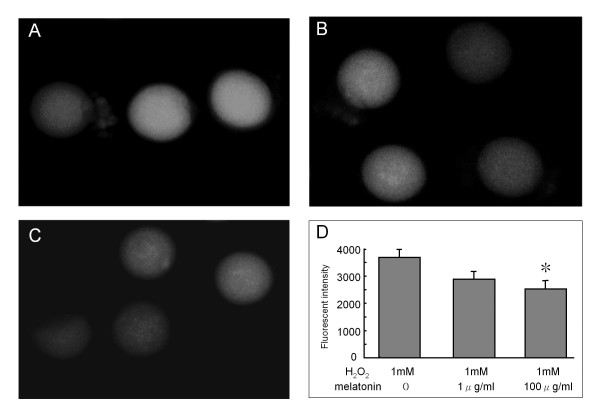
**The effect of melatonin on intracellular ROS production**. Immature (3 wks) ICR mice were given an injection of 20 units of pregnant mare serum gonadotropin (PMSG) to stimulate the development of multiple follicles. Oocytes were collected by puncturing ovarian follicles after 48 hrs PMSG injection, and then the surrounding cumulus cells were removed. Oocytes were incubated with 1 mM H_2_O_2 _for 10 min in the presence of melatonin (0, 1 μg/ml, 100 μg/ml). Intracellular ROS were detected using an intracellular dye (dichlorofuorescin:DCF-DA). The nonfluorescent DCF-DA is oxidized by intracellular ROS to form the highly fluorescent DCF, intracellular ROS formation was visualized by fluorescence image and fluorescence intensity was analyzed using MetaMorph software. (A) H_2_O_2 _(1 mM); (B) H_2_O_2 _(1 mM)+ melatonin (1 μg/ml); (C) H_2_O_2 _(1 mM)+ melatonin (100 μg/ml); (D) Fluorescence intensity in the oocytes. Data are shown as the mean ± SEM for (6-9 oocytes). *: p < 0.05 versus H_2_O_2 _(1 mM).

Oxidative stress in the oocyte caused by ROS must be limited in order for a good embryo to be produced. ROS induce lipid peroxidation of membranes and DNA damage in the oocyte and are expected to cause harmful effects in cell division, metabolite transport, and mitochondrial function [28]. We recently reported the direct effect of ROS and melatonin on oocyte maturation [57]. To investigate the effects of H_2_O_2 _on oocyte maturation, the denuded oocytes from immature mice treated with PMSG were cultured in the incubation medium with various concentrations of H_2_O_2_. After 12 hr incubation, oocytes with the first polar body (MII stage oocytes) were counted. The percentage of the mature oocytes (MII stage oocytes with a first polar body) was significantly decreased by the addition of H_2_O_2 _in a dose-dependent manner (> 200 μM). When oocytes were incubated with melatonin in the presence of H_2_O_2 _(300 μM), melatonin dose-dependently blocked the inhibitory effect of H_2_O_2 _on oocyte maturation, and there was a significant effect at the concentration of 10 ng/mL of melatonin. To further investigate the intra-cellular role of melatonin, oocytes were incubated with dichlorofluorescein (DCF-DA). The nonfluorescent DCF-DA was oxidized by intracellular ROS to form the highly fluorescent DCF, intracellular ROS formation was visualized by fluorescence image, and fluorescence intensity was analyzed. When oocytes were incubated without H_2_O_2_, there was no observable fluorescent intensity. However, high fluorescence intensities were observed in the presence of H_2_O_2 _(300 μm). The increased fluorescence intensity of oocytes incubated with H_2_O_2 _was significantly decreased by melatonin treatment (Figure 2). These results suggest that H_2_O_2 _inhibits oocytes maturation by producing ROS, but melatonin demonstrated protective activity against oxidative stress caused by H_2_O_2_. Recently, Kang et al., [58] investigated the effects of melatonin on the maturation of porcine oocytes. Oocytes from antral follicles were incubated in the medium with or without melatonin supplementation. Melatonin supplementation (10 ng/ml) during in-vitro maturation resulted in a greater proportion of oocytes extruding the polar body, and melatonin-treated oocytes had significantly lower levels of ROS than control (without melatonin treatment) oocytes.

**Figure 3:**
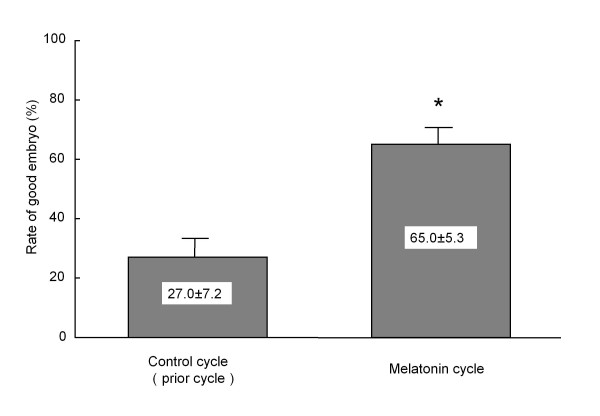
**Effect of melatonin treatment on embryo development in patients who underwent IVF-ET program**. Nine patients undergoing IVF-ET who failed to become pregnant in the prior IVF-ET cycle were given a 3 mg tablet of melatonin (KAL, Park City, UT, USA) orally at 22:00 hr from the fifth day of the previous menstrual cycle until the day of oocyte retrieval. Embryo quality was assessed according to Veeck criteria at 2 days after insemination, and Veeck I or II embryo was defined as a good embryo. Rate of good embryos was calculated; divide the number of good embryos (VeeckI or II) by the number of oocytes retrieved. Data are shown as the mean ± SEM. *: p < 0.05 versus control cycle.

The ability of melatonin to promote embryo development in different species has been reported. When inseminated mouse embryos were cultured in the medium with melatonin (10^-8^-10^-4 ^M), increased fertilization and blastocyst rates were observed [59]. Rodriguez-Osorio et al. [60] demonstrated the effects of melatonin on in-vitro porcine embryo development. Melatonin supplementation (10^-9 ^M) had a positive effect on the fertilization rates of inseminated porcine embryos that were cultured. Although blastocyst rates were not increased by melatonin, the number of blastocyst cells in the melatonin-supplemented group was significantly higher than in the control group. When the oocytes recovered from porcine follicles were incubated in the medium with melatonin (10^-7 ^M), fertilization rate, blastocyst rate and the number of blastocyst cells were significantly higher than that of the control (without melatonin) [61]. The effect of melatonin on embryo development seems to be, at least in part, caused by its action as an antioxidant, as Papis et al. [62] demonstrated that the beneficial effects of melatonin on bovine embryo development was observed not in a low oxygen environment but in a high oxygen environment where free radicals are easily produced. We recently confirmed the benefit of melatonin treatment to infertility women who underwent an IVF-ET program. When women were treated with 3 mg of melatonin daily from day 5 of the previous menstrual cycle until the day of oocyte retrieval, the percentage of good embryos (day 2 after insemination) was significantly higher compared to the control (without melatonin treatment) cycle (Figure 3). These data suggest that melatonin may be involved in oocyte maturation and embryo development.

### Clinical trial of melatonin for infertility patients

As summarized above, a growing amount of literature has demonstrated that melatonin and/or melatonin treatment may have a beneficial effect on oocyte maturation and embryo development. Poor oocyte quality is one of the most intractable causes of infertility in women. Melatonin treatment can be a useful infertility treatment and, therefore recently has been applied to infertility patients for the first time.

To document an association between melatonin and ovarian oxidative stress, human follicular fluids were sampled during oocyte retrieval for the purpose of IVF-ET and concentrations of melatonin and 8-OHdG were measured. The study revealed an inverse correlation between intra-follicular concentrations melatonin and 8-OHdG, suggesting that melatonin in the follicle diffuses into the cumulus and oocytes to protect them from free radical damage. When patients were given a 3 mg tablet of melatonin orally at 22:00 hr from the fifth day of the previous menstrual cycle until the day of oocyte retrieval, intra-follicular concentrations of melatonin rose from 112 pg/ml in the control cycle (without melatonin treatment) to 432 pg/ml after daily melatonin treatment. Intra-follicular concentrations of 8-OHdG and HEL, a damaged lipid product, were decreased after melatonin treatment compared to those in the prior cycle. The result demonstrates that melatonin treatment reduces intra-follicular oxidative damage. To investigate the clinical usefulness of melatonin administration, the effect of melatonin treatment on clinical outcome of IVF-ET was examined for 115 patients who failed to become pregnant in the previous IVF-ET cycle with a low fertilization rate (< 50%). In 56 patients with melatonin treatment, the fertilization rate (50.0 ± 38.0%) was markedly improved compared with the previous IVF-ET cycle (20.2 ± 19.0%), and 11 of 56 patients (19.6%) achieved pregnancy. On the other hand, in 59 patients who were not given melatonin, the fertilization rate (22.8 ± 19.0% vs 20.9 ± 16.5%) was not significantly changed, and only 6 of 59 patients (10.2%) achieved pregnancy. These results show that melatonin administration increases intra-follicular melatonin concentrations, reduces intra-follicular oxidative damage and elevates fertilization and pregnancy rates.

To our knowledge, our study represents the first clinical usefulness of melatonin treatment for infertility patients. Melatonin is likely to become a treatment for improving oocyte quality for women who cannot become pregnant because of poor oocyte quality.

## Conclusions

**Figure 4:**
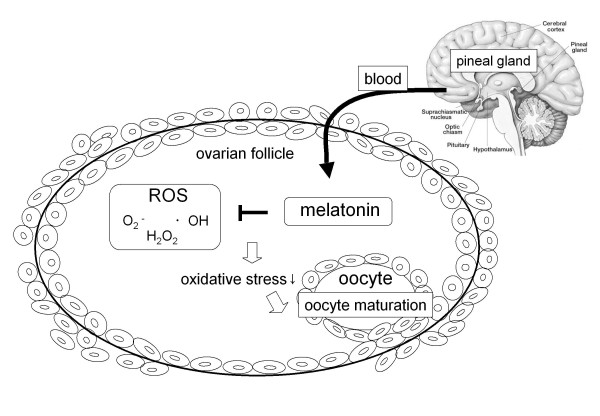
**Schematic representation of the presumed roles of melatonin in ovarian antral follicle**. Melatonin, secreted by pineal gland, is taken up into the follicular fluid from the blood. ROS produced within the follicles, especially ovulation process, were scavenged by melatonin, and reduced oxidative stress may be involved in oocyte maturation and embryo development.

The discovery of melatonin as a direct free radical scavenger has greatly broadened the understanding of its multiple physiological roles. The new findings regarding the potential role of melatonin in reproductive physiology have been also increasing. Melatonin is applicable to the regulation of seasonal reproductive events in photoperiod dependent breeding mammals, and it seems to be receptor mediated mechanism in hypothalamus and pituitary gland. However, recently many researchers have begun to study the local role of melatonin as an antioxidant. We focused on intra-follicular role of melatonin in the ovary. Melatonin, secreted by pineal gland, is taken up into the follicular fluid from the blood. ROS produced within the follicles, especially during the ovulation process, were scavenged by melatonin, and reduced oxidative stress may be involved in oocyte maturation and embryo development (Figure 4). Our clinical study demonstrated that melatonin treatment for infertility women increases intra-follicular melatonin concentrations, reduces intra-follicular oxidative damage and elevates fertilization and pregnancy rates. It should be noted that melatonin treatment could become a new cure for improving oocyte quality in infertility patients, this is consistent with the most detailed review which was reported recently [63]. The safety of exogenous melatonin for humans has been shown in many studies [64,65]. It has also been reported that melatonin have no detrimental effects on mouse and rat embryo development during toxicity tests that were performed both in-vitro and in-vivo [58,66,67].

## Competing interests

The authors declare that they have no competing interests.

## Authors' contributions

HT participated in drafting the full manuscript and writing of this manuscript. AT clinical study design, coordination and data analysis. TT and MT performed the mouse oocyte experiments and analyzed the data. FK and LL participated in creating figures. IT and RM contributed by writing specific sections of this manuscript. HA and YY provided advice and participated in revising the manuscript. NS participated in substantial contribution to conception and revising it critically for important intellectual content. All the authors in this manuscript have read and approved the final version.

## References

[B1] Brigelius-Flohe R Banning A Kny M Bol GF Redox events in interleukin-1 signaling Arch Biochem Biophys 2004 423 1 66 73 10.1016/j.abb.2003.12.008 14989266

[B2] Forman HJ Torres M Reactive oxygen species and cell signaling: respiratory burst in macrophage signaling Am J Respir Crit Care Med 2002 166 12 Pt 2 S4 8 12471082 10.1164/rccm.2206007

[B3] Li Q Sanlioglu S Li S Ritchie T Oberley L Engelhardt JF GPx-1 gene delivery modulates NFkappaB activation following diverse environmental injuries through a specific subunit of the IKK complex Antioxid Redox Signal 2001 3 3 415 432 10.1089/15230860152409068 11491654

[B4] Agarwal A Gupta S Sharma RK Role of oxidative stress in female reproduction Reprod Biol Endocrinol 2005 3 28 10.1186/1477-7827-3-28 16018814 PMC1215514

[B5] Goud AP Goud PT Diamond MP Gonik B Abu-Soud HM Reactive oxygen species and oocyte aging: role of superoxide, hydrogen peroxide, and hypochlorous acid Free Radic Biol Med 2008 44 7 1295 1304 10.1016/j.freeradbiomed.2007.11.014 18177745 PMC3416041

[B6] Yang HW Hwang KJ Kwon HC Kim HS Choi KW Oh KS Detection of reactive oxygen species (ROS) and apoptosis in human fragmented embryos Hum Reprod 1998 13 4 998 1002 10.1093/humrep/13.4.998 9619561

[B7] Gillette MU Tischkau SA Suprachiasmatic nucleus: the brain's circadian clock Recent Prog Horm Res 1999 54 33 58; discussion 58-39 10548871

[B8] Reiter RJ Pineal melatonin: cell biology of its synthesis and of its physiological interactions Endocr Rev 1991 12 2 151 180 10.1210/edrv-12-2-151 1649044

[B9] Cardinali DP Pevet P Basic aspects of melatonin action Sleep Med Rev 1998 2 3 175 190 10.1016/S1087-0792(98)90020-X 15310500

[B10] Reiter RJ Tan DX Korkmaz A The circadian melatonin rhythm and its modulation: possible impact on hypertension J Hypertens Suppl 2009 27 6 S17 20 10.1097/01.hjh.0000358832.41181.bf 19633446

[B11] Srinivasan V Maestroni GJ Cardinali DP Esquifino AI Perumal SR Miller SC Melatonin, immune function and aging Immun Ageing 2005 2 17 10.1186/1742-4933-2-17 16316470 PMC1325257

[B12] Korkmaz A Tamura H Manchester LC Ogden GB Tan DX Reiter RJ Combination of melatonin and a peroxisome proliferator-activated receptor-gamma agonist induces apoptosis in a breast cancer cell line J Pineal Res 2009 46 1 115 116 10.1111/j.1600-079X.2008.00635.x 18798787

[B13] Tamura H Nakamura Y Narimatsu A Yamagata Y Takasaki A Reiter RJ Sugino N Melatonin treatment in peri- and postmenopausal women elevates serum high-density lipoprotein cholesterol levels without influencing total cholesterol levels J Pineal Res 2008 45 1 101 105 10.1111/j.1600-079X.2008.00561.x 18298467

[B14] Takayama H Nakamura Y Tamura H Yamagata Y Harada A Nakata M Sugino N Kato H Pineal gland (melatonin) affects the parturition time, but not luteal function and fetal growth, in pregnant rats Endocr J 2003 50 1 37 43 10.1507/endocrj.50.37 12733707

[B15] Tamura H Nakamura Y Terron MP Flores LJ Manchester LC Tan DX Sugino N Reiter RJ Melatonin and pregnancy in the human Reprod Toxicol 2008 25 3 291 303 10.1016/j.reprotox.2008.03.005 18485664

[B16] Tamura H Takayama H Nakamura Y Reiter RJ Sugino N Fetal/placental regulation of maternal melatonin in rats J Pineal Res 2008 44 3 335 340 10.1111/j.1600-079X.2007.00537.x 18339129

[B17] Tamura H Nakamura Y Takiguchi S Kashida S Yamagata Y Sugino N Kato H Pinealectomy of melatonin implantation does not affect prolactin surge or luteal function in pseudopregnant rats Endocr J 1998 45 3 377 383 10.1507/endocrj.45.377 9790273

[B18] Brzezinski A Seibel MM Lynch HJ Deng MH Wurtman RJ Melatonin in human preovulatory follicular fluid J Clin Endocrinol Metab 1987 64 4 865 867 10.1210/jcem-64-4-865 3818907

[B19] Ronnberg L Kauppila A Leppaluoto J Martikainen H Vakkuri O Circadian and seasonal variation in human preovulatory follicular fluid melatonin concentration J Clin Endocrinol Metab 1990 71 2 492 496 2380343 10.1210/jcem-71-2-493

[B20] Nakamura Y Tamura H Takayama H Kato H Increased endogenous level of melatonin in preovulatory human follicles does not directly influence progesterone production Fertil Steril 2003 80 4 1012 1016 10.1016/S0015-0282(03)01008-2 14556825

[B21] Brannstrom M Norman RJ Involvement of leukocytes and cytokines in the ovulatory process and corpus luteum function Hum Reprod 1993 8 10 1762 1775 8300842 10.1093/oxfordjournals.humrep.a137929

[B22] Nakamura Y Smith M Krishna A Terranova PF Increased number of mast cells in the dominant follicle of the cow: relationships among luteal, stromal, and hilar regions Biol Reprod 1987 37 3 546 549 10.1095/biolreprod37.3.546 3676403

[B23] Droge W Free radicals in the physiological control of cell function Physiol Rev 2002 82 1 47 95 11773609 10.1152/physrev.00018.2001

[B24] Hensley K Robinson KA Gabbita SP Salsman S Floyd RA Reactive oxygen species, cell signaling, and cell injury Free Radic Biol Med 2000 28 10 1456 1462 10.1016/S0891-5849(00)00252-5 10927169

[B25] Fatehi AN Roelen BA Colenbrander B Schoevers EJ Gadella BM Beverst MM van den Hurk R Presence of cumulus cells during in vitro fertilization protects the bovine oocyte against oxidative stress and improves first cleavage but does not affect further development Zygote 2005 13 2 177 185 10.1017/S0967199405003126 16128413

[B26] Tatemoto H Muto N Sunagawa I Shinjo A Nakada T Protection of porcine oocytes against cell damage caused by oxidative stress during in vitro maturation: role of superoxide dismutase activity in porcine follicular fluid Biol Reprod 2004 71 4 1150 1157 10.1095/biolreprod.104.029264 15175235

[B27] Zuelke KA Jones DP Perreault SD Glutathione oxidation is associated with altered microtubule function and disrupted fertilization in mature hamster oocytes Biol Reprod 1997 57 6 1413 1419 10.1095/biolreprod57.6.1413 9408248

[B28] Guerin P El Mouatassim S Menezo Y Oxidative stress and protection against reactive oxygen species in the pre-implantation embryo and its surroundings Hum Reprod Update 2001 7 2 175 189 10.1093/humupd/7.2.175 11284661

[B29] Kowaltowski AJ Vercesi AE Mitochondrial damage induced by conditions of oxidative stress Free Radic Biol Med 1999 26 3-4 463 471 10.1016/S0891-5849(98)00216-0 9895239

[B30] Noda Y Matsumoto H Umaoka Y Tatsumi K Kishi J Mori T Involvement of superoxide radicals in the mouse two-cell block Mol Reprod Dev 1991 28 4 356 360 10.1002/mrd.1080280408 1648368

[B31] Chao HT Lee SY Lee HM Liao TL Wei YH Kao SH Repeated ovarian stimulations induce oxidative damage and mitochondrial DNA mutations in mouse ovaries Ann N Y Acad Sci 2005 1042 148 156 10.1196/annals.1338.016 15965057

[B32] Al-Gubory KH Bolifraud P Germain G Nicole A Ceballos-Picot I Antioxidant enzymatic defence systems in sheep corpus luteum throughout pregnancy Reproduction 2004 128 6 767 774 10.1530/rep.1.00389 15579594

[B33] Paszkowski T Traub AI Robinson SY McMaster D Selenium dependent glutathione peroxidase activity in human follicular fluid Clin Chim Acta 1995 236 2 173 180 10.1016/0009-8981(95)98130-9 7554284

[B34] Tarin JJ Perez-Albala S Cano A Oral antioxidants counteract the negative effects of female aging on oocyte quantity and quality in the mouse Mol Reprod Dev 2002 61 3 385 397 10.1002/mrd.10041 11835584

[B35] Poeggeler B Reiter RJ Tan DX Chen LD Manchester LC Melatonin, hydroxyl radical-mediated oxidative damage, and aging: a hypothesis J Pineal Res 1993 14 4 151 168 10.1111/j.1600-079X.1993.tb00498.x 8102180

[B36] Schindler AE Christensen B Henkel A Oettel M Moore C High-dose pilot study with the novel progestogen dienogestin patients with endometriosis Gynecol Endocrinol 2006 22 1 9 17 10.1080/09513590500431482 16522528

[B37] Reiter RJ Tan DX Manchester LC Qi W Biochemical reactivity of melatonin with reactive oxygen and nitrogen species: a review of the evidence Cell Biochem Biophys 2001 34 2 237 256 10.1385/CBB:34:2:237 11898866

[B38] Tan DX Manchester LC Reiter RJ Plummer BF Limson J Weintraub ST Qi W Melatonin directly scavenges hydrogen peroxide: a potentially new metabolic pathway of melatonin biotransformation Free Radic Biol Med 2000 29 11 1177 1185 10.1016/S0891-5849(00)00435-4 11121726

[B39] Peyrot F Ducrocq C Potential role of tryptophan derivatives in stress responses characterized by the generation of reactive oxygen and nitrogen species J Pineal Res 2008 45 3 235 246 10.1111/j.1600-079X.2008.00580.x 18341517

[B40] Tan DX Manchester LC Terron MP Flores LJ Reiter RJ One molecule, many derivatives: a never-ending interaction of melatonin with reactive oxygen and nitrogen species? J Pineal Res 2007 42 1 28 42 10.1111/j.1600-079X.2006.00407.x 17198536

[B41] Hardeland R Antioxidative protection by melatonin: multiplicity of mechanisms from radical detoxification to radical avoidance Endocrine 2005 27 2 119 130 10.1385/ENDO:27:2:119 16217125

[B42] Allegra M Reiter RJ Tan DX Gentile C Tesoriere L Livrea MA The chemistry of melatonin's interaction with reactive species J Pineal Res 2003 34 1 1 10 10.1034/j.1600-079X.2003.02112.x 12485365

[B43] Reiter RJ Tan DX Gitto E Sainz RM Mayo JC Leon J Manchester LC Vijayalaxmi Kilic E Kilic U Pharmacological utility of melatonin in reducing oxidative cellular and molecular damage Pol J Pharmacol 2004 56 2 159 170 15156066

[B44] Tan DX Manchester LC Sainz RM Mayo JC Leon J Hardeland R Poeggeler B Reiter RJ Interactions between melatonin and nicotinamide nucleotide: NADH preservation in cells and in cell-free systems by melatonin J Pineal Res 2005 39 2 185 194 10.1111/j.1600-079X.2005.00234.x 16098097

[B45] Silva SO Rodrigues MR Carvalho SR Catalani LH Campa A Ximenes VF Oxidation of melatonin and its catabolites, N1-acetyl-N2 -formyl-5-methoxykynuramine and N1-acetyl-5-methoxykynuramine, by activated leukocytes J Pineal Res 2004 37 3 171 175 10.1111/j.1600-079X.2004.00149.x 15357661

[B46] Manda K Ueno M Anzai K AFMK, a melatonin metabolite, attenuates X-ray-induced oxidative damage to DNA, proteins and lipids in mice J Pineal Res 2007 42 4 386 393 10.1111/j.1600-079X.2007.00432.x 17439555

[B47] Rosen J Than NN Koch D Poeggeler B Laatsch H Hardeland R Interactions of melatonin and its metabolites with the ABTS cation radical: extension of the radical scavenger cascade and formation of a novel class of oxidation products, C2-substituted 3-indolinones J Pineal Res 2006 41 4 374 381 10.1111/j.1600-079X.2006.00379.x 17014695

[B48] Mayo JC Sainz RM Antoli I Herrera F Martin V Rodriguez C Melatonin regulation of antioxidant enzyme gene expression Cell Mol Life Sci 2002 59 10 1706 1713 10.1007/PL00012498 12475181 PMC11337431

[B49] Rodriguez C Mayo JC Sainz RM Antolin I Herrera F Martin V Reiter RJ Regulation of antioxidant enzymes: a significant role for melatonin J Pineal Res 2004 36 1 1 9 10.1046/j.1600-079X.2003.00092.x 14675124

[B50] Chen LJ Gao YQ Li XJ Shen DH Sun FY Melatonin protects against MPTP/MPP+ -induced mitochondrial DNA oxidative damage in vivo and in vitro J Pineal Res 2005 39 1 34 42 10.1111/j.1600-079X.2005.00209.x 15978055

[B51] Melchiorri D Reiter RJ Sewerynek E Chen LD Nistico G Melatonin reduces kainate-induced lipid peroxidation in homogenates of different brain regions FASEB J 1995 9 12 1205 1210 7672513 10.1096/fasebj.9.12.7672513

[B52] Ortega-Gutierrez S Fuentes-Broto L Garcia JJ Lopez-Vicente M Martinez-Ballarin E Miana-Mena FJ Millan-Plano S Reiter RJ Melatonin reduces protein and lipid oxidative damage induced by homocysteine in rat brain homogenates J Cell Biochem 2007 102 3 729 735 10.1002/jcb.21327 17427950

[B53] Tan DX Manchester LC Reiter RJ Cabrera J Burkhardt S Phillip T Gitto E Karbownik M Li QD Melatonin suppresses autoxidation and hydrogen peroxide-induced lipid peroxidation in monkey brain homogenate Neuro Endocrinol Lett 2000 21 5 361 365 11452230

[B54] Yamamoto HA Mohanan PV Melatonin attenuates brain mitochondria DNA damage induced by potassium cyanide in vivo and in vitro Toxicology 2002 179 1-2 29 36 10.1016/S0300-483X(02)00244-5 12204540

[B55] Wurtman RJ Axelrod J Potter LT The uptake of h3-melatonin in endocrine and nervous tissues and the effects of constant light exposure J Pharmacol Exp Ther 1964 143 314 318 14161142

[B56] Tamura H Nakamura Y Takiguchi S Kashida S Yamagata Y Sugino N Kato H Melatonin directly suppresses steroid production by preovulatory follicles in the cyclic hamster J Pineal Res 1998 25 3 135 141 10.1111/j.1600-079X.1998.tb00551.x 9745981

[B57] Tamura H Takasaki A Miwa I Taniguchi K Maekawa R Asada H Taketani T Matsuoka A Yamagata Y Shimamura K Oxidative stress impairs oocyte quality and melatonin protects oocytes from free radical damage and improves fertilization rate J Pineal Res 2008 44 3 280 287 10.1111/j.1600-079X.2007.00524.x 18339123

[B58] Jahnke G Marr M Myers C Wilson R Travlos G Price C Maternal and developmental toxicity evaluation of melatonin administered orally to pregnant Sprague-Dawley rats Toxicol Sci 1999 50 2 271 279 10.1093/toxsci/50.2.271 10478864

[B59] Ishizuka B Kuribayashi Y Murai K Amemiya A Itoh MT The effect of melatonin on in vitro fertilization and embryo development in mice J Pineal Res 2000 28 1 48 51 10.1034/j.1600-079x.2000.280107.x 10626601

[B60] Rodriguez-Osorio N Kim IJ Wang H Kaya A Memili E Melatonin increases cleavage rate of porcine preimplantation embryos in vitro J Pineal Res 2007 43 3 283 288 10.1111/j.1600-079X.2007.00475.x 17803526

[B61] Shi JM Tian XZ Zhou GB Wang L Gao C Zhu SE Zeng SM Tian JH Liu GS Melatonin exists in porcine follicular fluid and improves in vitro maturation and parthenogenetic development of porcine oocytes J Pineal Res 2009 47 4 318 323 10.1111/j.1600-079X.2009.00717.x 19817971

[B62] Papis K Poleszczuk O Wenta-Muchalska E Modlinski JA Melatonin effect on bovine embryo development in vitro in relation to oxygen concentration J Pineal Res 2007 43 4 321 326 10.1111/j.1600-079X.2007.00479.x 17910599

[B63] Reiter RJ Tan DX Manchester LC Paredes SD Mayo JC Sainz RM Melatonin and reproduction revisited Biol Reprod 2009 81 3 445 456 10.1095/biolreprod.108.075655 19439728

[B64] Buscemi N Vandermeer B Hooton N Pandya R Tjosvold L Hartling L Vohra S Klassen TP Baker G Efficacy and safety of exogenous melatonin for secondary sleep disorders and sleep disorders accompanying sleep restriction: meta-analysis BMJ 2006 332 7538 385 393 10.1136/bmj.38731.532766.F6 16473858 PMC1370968

[B65] Carr R Wasdell MB Hamilton D Weiss MD Freeman RD Tai J Rietveld WJ Jan JE Long-term effectiveness outcome of melatonin therapy in children with treatment-resistant circadian rhythm sleep disorders J Pineal Res 2007 43 4 351 359 10.1111/j.1600-079X.2007.00485.x 17910603

[B66] Chan WY Ng TB Development of pre-implantation mouse embryos under the influence of pineal indoles J Neural Transm Gen Sect 1994 96 1 19 29 10.1007/BF01277925 7531981

[B67] McElhinny AS Davis FC Warner CM The effect of melatonin on cleavage rate of C57BL/6 and CBA/Ca preimplantation embryos cultured in vitro J Pineal Res 1996 21 1 44 48 10.1111/j.1600-079X.1996.tb00269.x 8836963

